# Activation of GluN2D-containing NMDA receptors promotes development of axons and axon-carrying dendrites of cortical interneurons

**DOI:** 10.1093/cercor/bhaf136

**Published:** 2025-06-06

**Authors:** Ina Köhler, Lisa-Marie Rennau, Leon Hoffmann, Ekaterina Demianchuk, Michelle Kaczmarski, Eric Sobierajski, Christian Riedel, Petra Wahle

**Affiliations:** Developmental Neurobiology, Faculty of Biology and Biotechnology, Ruhr University Bochum, Universitätsstraße 150, Bochum 44780, Germany; Developmental Neurobiology, Faculty of Biology and Biotechnology, Ruhr University Bochum, Universitätsstraße 150, Bochum 44780, Germany; Developmental Neurobiology, Faculty of Biology and Biotechnology, Ruhr University Bochum, Universitätsstraße 150, Bochum 44780, Germany; Developmental Neurobiology, Faculty of Biology and Biotechnology, Ruhr University Bochum, Universitätsstraße 150, Bochum 44780, Germany; Developmental Neurobiology, Faculty of Biology and Biotechnology, Ruhr University Bochum, Universitätsstraße 150, Bochum 44780, Germany; Developmental Neurobiology, Faculty of Biology and Biotechnology, Ruhr University Bochum, Universitätsstraße 150, Bochum 44780, Germany; Developmental Neurobiology, Faculty of Biology and Biotechnology, Ruhr University Bochum, Universitätsstraße 150, Bochum 44780, Germany

**Keywords:** axogenesis, dendritogenesis, fast-spiking and regular-spiking inhibitory neurons, glutamate receptor, interneuronal proteins

## Abstract

GluN2D-containing NMDA receptors are expressed in early postnatal interneurons, but their role is enigmatic. We tested whether treatment with the GluN2C/D positive allosteric modulator CIQ and non-competitive antagonist DQP-1105 from days in vitro (DIV) 5–10 and DIV 15–20 modulates neurite growth in organotypic cultures. Calcium imaging confirmed a functional expression of GluN2D in nonpyramidal neurons. DQP treatment enhanced apical dendritic branching and increased ERK1/2 phosphorylation and spine density, suggesting a disinhibitory effect mirrored by a reduced expression of GAD-65, VGAT, and Syt-2. Control basket cells had larger axon-carrying dendrites (AcDs), and under CIQ, the AcDs grew even larger. The axons of CIQ-treated basket cells formed more branches within the dendritic field, and the effect was strongest for axons emerging from AcDs. DQP-treated basket cells also displayed more complex AcDs, presumably driven by enhanced network activity. However, local branching of basket cell axons was reduced under DQP in somatic axon cells but at control level in AcD cells. This suggested a growth-promoting effect of the enhanced network activity and that the AcD configuration neutralized the inhibitory action of DQP on basket cell axons. The results suggest a specific role of GluN2D signaling for development and remodeling of interneuronal axons.

## Introduction

The maturation of interneurons depends on their genetic program, neuronal activity, and environmental cues. In rodent cortex, calcium transients detected already at embryonic day 16 become highly synchronized perinatally, spreading over large areas of the cortex (Corlew et al. 2004; Allène et al. 2008; Luhmann et al. 2016). These early activity patterns are mediated by glutamatergic signaling, and AMPA, kainate, and NMDA-type receptors play essential roles. Neurotrophin signaling acts in conjunction with activity to promote the morphological maturation of cortical pyramidal cells and, in particular, interneurons (McAllister et al. 1995; Gorba and Wahle 1999; Wirth et al. 2003; Jin et al. 2003; Engelhardt et al. 2007). Blocking glutamate receptors early in development with kynurenic acid causes, at postnatal day (P) 8–9, a reduced axonal length and complexity of calretinin- and reelin-positive interneurons (Marco García et al. 2011). Biolistic overexpression in organotypic cultures of the AMPA receptor GluA1Q-flip splice variant increases interneuronal dendritic length and branching (Hamad et al. 2011). Overexpression of the GluK1 kainate receptor subunit in organotypic cultures promotes dendritic growth of interneurons at days in vitro (DIV) 10 (Jack et al. 2019). Inhibiting NMDA receptors with APV in tadpole tectal neurons reduces dendritic development (Rajan and Cline 1998). Similarly, blocking NMDA receptors in vivo from P1–21 impairs dendritic development in rodent cortex (Wedzony et al. 2005). In line, blocking of GluN2B-containing, but not GluN2A-containing receptors in organotypic cultures from DIV1–10 impairs the growth of visual cortex pyramidal basal dendrites, but does not affect interneurons (Gonda et al. 2020).

GluN2D receptors differ from other GluN2 receptors. Deactivation time is 10 times slower compared to GluN2B/2C, and 100 times slower than that of GluN2A subunits (Vance et al. 2011). The Mg^2+^ block is 10 times weaker (Qian et al. 2005; Clarke and Johnson 2006). Therefore, GluN2D receptors do not require a large AMPA-mediated pre-depolarization to activate. Additionally, GluN2C/2D receptors have a higher affinity for glutamate and can activate at relatively low glutamate concentrations (Hollmann and Heinemann 1994). GluN2D in the CNS is associated with GluN1 and GluN2A/2B, suggestive of di- or triheteromeric receptors (Dunah et al. 1998).

Investigation of GluN2C/2D-containing NMDA receptors relies on compounds such as the positive allosteric modulator CIQ and the non-competitive antagonist DQP-1105 (DQP in the following). CIQ binds to the M1 region of the GluN2C/2D receptor and modulates the opening time without affecting permeability (Ogden and Traynelis 2013). Activation with CIQ increases the amplitude of action potentials of parvalbumin-positive basket neurons by 180% (Mullasseril et al. 2010; Garst et al. 2020). DQP hinders glutamate and glycine from binding to the receptor and can change the activity of cortical neurons early in development (Acker et al. 2011).

GluN2C is mainly in astrocytes (Alsaad et al. 2019). GluN2D is expressed in telencephalic interneurons with higher abundance in fast-spiking basket cells (basket cells) containing parvalbumin but also in non-fast-spiking basket cells containing cholecystokinin (Booker et al. 2021). The non-basket cells (non-basket cells) expressing e.g. somatostatin or calretinin have less GluN2D, and, except for a brief transient perinatal expression, GluN2D is absent in pyramidal cells (Von Engelhardt et al. 2015; Alsaad et al. 2019; Garst et al. 2020). Not much is known about the developmental role of GluN2D. Perinatally, the highest GluN2D protein levels are at P7 in rat thalamus and midbrain (Dunah et al. 1996; Swanger et al. 2015). Comparing the roles of GluN2B to GluN2D in the ascending whisker-to-barrel pathway has revealed that reducing the amount of GluN2B subunits delays, whereas knockout of GluN2D advances by about 1 day the formation of barrels in brainstem, thalamus, and somatosensory cortex. Thus, GluN2D localized at excitatory synapses onto GAD-67-positive cells along the pathway acts indirectly to slow down formation or refinement of the projection (Yamasaki et al. 2014). Also in the neocortex, GluN2D expression peaks at P7–10 and declines until P40 (Akazawa et al. 1994; Monyer et al. 1994; Standaert et al. 1994; Dunah et al. 1996). In the hippocampus, GluN2D-containing receptors contribute to the activation of interneurons (Perszyk et al. 2016; Swanger et al. 2018), and the increased charge transfer enhances the inhibitory output of interneurons to pyramidal cells, which themselves are not affected by GluN2D activators (Camp et al. 2024). For instance, in the sensorimotor cortex, it has been reported that the pharmacological blockade of GluN2D receptors with DQP injected intraperitoneally at 5 mM at P7–9 alters a transient, tonic NMDA current and leads to a reduced morphological complexity of parvalbuminergic basket cells at P21 as revealed by reconstruction of biocytin-filled fast-spiking neurons (Hanson et al. 2019).

Knowledge of the developmental function of GluN2D-containing receptors is of clinical importance. Mutations of NMDA receptors lead to a host of neuropathological conditions, with childhood epileptic encephalopathy being of particular relevance. Carriers of GluN2D mutations present with heterogeneous symptoms and often do not respond to anti-epilepsy medications (Camp and Yuan 2020). GluN2D gain-of-function mutations have been described with higher sensitivity to agonists, increased channel open time, and reduced sensitivity to endogenous negative modulators, and the progressive loss of interneurons with hyperactivated NMDA receptors leads to intractable childhood epileptic encephalopathy. Introducing mutated GluN2D subunits into cultured neurons causes severe dendritic injury and neuronal death, whereas channel blockers administered to the patient ameliorated the seizure burden (Li et al. 2016). Loss-of-function mutations also result in epilepsies because they impair the proper activation of inhibitory neurons. Equally important is the potential to enhance circuit inhibition in psychiatric diseases, and a novel positive modulator of GluN2D has been shown to promote gamma oscillations (Camp et al. 2024). Recently, GluN1/2D/2B triheteromeric receptors have been reported to be the target of ketamine used to relieve depression (Kang et al. 2025). Accordingly, GluN2D activators and blockers are under investigation to treat mental and neurodegenerative disorders, recognizing that many disorders have a neurodevelopmental origin. For instance, CIQ increases the excitability of parvalbumin-positive basket cells, and does so also in a mouse model for schizophrenia with hypoexcitable basket cells (Garst et al. 2020). CIQ-enhanced NMDA receptor activation has shown promise for treating Parkinson’s disease (Nouhi et al. 2018). These observations suggest that GluN2D-containing receptors are important for healthy postnatal development and, when mutated, pave the way to pathology. Here, we used CIQ and DQP directly in organotypic slice cultures to test if modulation of GluN2D receptor-mediated activity could alter the morphological complexity of visual cortical pyramidal cells and inhibitory neurons.

## Material and methods

### Ethic statement

All experiments were conducted in accordance with National and European (2010/63/EU) laws for the use of animals in research. Neonatal rats, born and housed in the local animal facility, were used for organotypic slice cultures. According to the German Animal Welfare Act (“Tierschutzgesetz der Bundesrepublik Deutschland, TierSchG”, § 4), the killing of vertebrate animals in order to remove tissue or organs for scientific purposes does not require official or institutional authorization. Therefore, no file number has been assigned to this project.

### Organotypic cultures

Roller-type organotypic cultures were prepared from pigmented Long-Evans rats as described. Briefly, the visual cortex was explanted at P0–2 (P0 day of birth, 5–6 pups per preparation) and chopped into 350 μm-thick slices with a McIlwain tissue chopper. Slices from every individual were allocated to the control, CIQ-, and DQP-treated groups. Cultures were kept in a culture medium containing 10% adult horse serum, 25% Hank’s balanced Salt Solution, 50% Eagle’s Basal Medium, 1 mM L-glutamine (all from Life Technologies, Karlsruhe, Germany), and 0.065% D-glucose (Merck, Darmstadt, Germany). The medium was changed every two days. To inhibit excessive glial growth, a mixture of anti-mitotic inhibitors (uridine, cytosine-ß-D-arabinofuranoside, and 5-fluorodeoxyuridine, all 10 μM final concentration, all from Sigma-Aldrich) was given at DIV 1 and removed after 24 hours with a medium change.

### Plasmids, biolistic transfection, and pharmacological treatment

Plasmids (Table 1) were prepared as endotoxin-free solutions using the EndoFree Plasmid Maxi Kit (Qiagen, Hilden, Germany, cat no. 12362). Plasmid stocks were diluted to 1 μg/μl and stored at −20°C. Cultures were transfected with a Gene Gun (Helios, BioRad, Munich, Germany) as described (Hamad et al. 2011; Gasterstädt et al. 2022; Gonda et al. 2023a). Briefly, cartridges were prepared by coating 7 mg gold microparticles (diameter 1 μm; BioRad, Munich, Germany) with 10 μg plasmid DNA. Cultures were blasted at 180 psi helium pressure at DIV 4 with morphological assessment at DIV 10. For the second time window, transfection was performed at DIV 14 with analysis at DIV 20. CIQ (3-chlorophenyl)(6,7-dimethoxy-1-((4-methoxyphenoxy)-methyl)-3,4-dihydroisoquinolin-2(1*H*)-yl)methanone) and DQP (4-(5-(4-bromophenyl)-3-(6-methyl-2-oxo-4-phenyl-1,2-dihydroquinolin-3-yl)-4,5-dihydro-1H-pyrazol-1-yl)-4-oxobutanoic acid) (both from Tocris, Wiesbaden-Nordenstadt, Germany) were dissolved in dimethylsulfoxide (DMSO) and stocks were stored frozen. Drugs were applied at 15 μM final concentration in the medium from DIV 5–10 or DIV 15–20 daily, with a medium change every second day. Control cultures received the same amount of DMSO in water. The total DMSO concentration in the medium did not exceed 0.01%.

**Table 1:** Plasmids, antibodies, and reagents.

Material	Species	Source	cat. No., RRID	Method, Dilution	Antigen (kDa)
Plasmids					
pEGFP-N1 in pcDNA3.0	–	Clontech	632370	–	–
pAAV-mDlx-GCaMP6f-Fishell-2	–	Dimidschstein et al. 2016 via Addgene	RRID: Addgene_83899	–	–
pAAV-mDlx-GFP-Fishell-1	–	Dimidschstein et al. 2016 via Addgene	RRID: Addgene_83900	–	–
Primary antibodies					
EGFP	Mouse	Merck	clone GSN24 RRID: AB_563117	IHC, 1:1000	–
β-actin	Mouse	Merck	A1978 RRID: AB_476692	WB, 1:1000	40
βIII-tubulin	Mouse	Merck	T8660 RRID: AB_477590	WB, 1:10000	55
GluN2D	Mouse	Merck	MAB5578 RRID: AB_838227	WB, 1:1000	150
GluN2D	Rabbit	BiCell Scientific	15145	IHC, 1:1000	150
PSD 95	Rabbit	Synaptic Systems,	124 011 RRID: AB_10804286	WB, 1:1000	95
GluN2B	Rabbit	Merck	06–600 RRID: AB_310193	WB, 1:1000	180
GAD 65/67	Mouse	Enzo Life Sciences	ADI-MSA-225E RRID: AB_2039129	WB, 1:1500	65/67
Synaptotagmin-2	Rabbit	Synaptic Systems	105 225, RRID: AB_2744654	WB, 1:1000	70
VGAT (cytosolic domain)	Rabbit	Synaptic Systems	131 002 RRID: AB_887871	WB 1:1000	57
Kv3.1b	Rabbit	Synaptic Systems	242 003 RRID: AB_11043175	WB 1:1000	110
Synaptophysin 1	Mouse	Synaptic Systems	101 011 RRID: AB_887824	WB, 1:1000	38
Synapsin-1	Mouse	Synaptic Systems	106 011, RRID: AB_2619772	WB, 1:2000	78
GABA(A)R α1	Mouse	NeuroMab	75–136 RRID: AB_ 2,877,288	WB, 1:700	52
ERK 1/2	Mouse	Santa Cruz Biotechnology	Sc-135,900 RRID: AB_2141283	WB, 1:1000	44/42
phospho-ERK 1/2	Mouse	Merck	M8159 RRID: AB_477245	WB, 1:1000	44/42
phospho-ERK 1/2	Rabbit	New England Biolabs	9101S	WB, 1:1000	44/42
GFAP	Rabbit	DAKO A/S	Z0334 RRID: AB_10013382	WB 1:1000	50
Secondary antibodies					
anti-rabbit biotinylated	Goat	Dako A/S	E0432 RRID: AB_2313609	IHC, 1:750	–
anti-mouse biotinylated	Goat	Dako A/S	E0433 RRID: AB_2687905	IHC, 1:750	–
anti-mouse Alexa 488	Goat	Thermo-Fischer Scientific	A-11001 RRID: AB_2534069	IHC, 1:1000	–
anti-mouse-AP	Rabbit	Dako A/S	D0314	WB, 1:5000	–
anti-rabbit-AP	Goat	Dako A/S	D0487 RRID: AB_2617144	WB, 1:2000	–
Reagents					
ABC reagent		Vector Laboratories Inc.	PK-7100 RRID: AB_2336827	as recommended	–

### Immunostaining

Cultures were fixed with 4% paraformaldehyde in 0.1 M phosphate buffer (PB) for 60 min and rinsed with PB. Permeabilization was for 2 hours with 0.5% Triton X-100 in PB, followed by washes, blocking with 5% BSA in TBS for 60 min, primary antibody incubation mouse anti-EGFP or rabbit anti-GluN2D for 12–24 h, followed by biotinylated secondary overnight, a 2 h incubation with ABC reagent, and HRP reaction with 0.01% diaminobenzidine (Sigma Aldrich, Steinheim, Germany) and 0.005% final concentration of H_2_O_2_. The reaction product was intensified with 1% OsO_4_ (Sigma Aldrich, Steinheim, Germany) in phosphate buffer for 30 sec. Cultures were dehydrated and coverslipped in DEPEX (Sigma Aldrich, Steinheim, Germany).

### Protein blots

Lysates and blots were performed as described (Engelhardt et al. 2018) with cultures derived from > 10 preparations. Two samples of rat visual cortex in vivo were taken from our frozen tissue collection. For ERK1/2 phosphorylation, cultures at DIV 10 and DIV 20 were acutely exposed for 30 minutes to CIQ or DQP (15 μM each) and lysed with RIPA-SDS buffer. Lysates (of 1 culture per lane) were separated on 10% SDS-PAGE gels. Membrane strips were placed over the molecular weight position of each protein of interest, which allowed the assessment of several proteins with distinct kDa in every lysate (Engelhardt et al. 2018). The densitometric intensity of the target protein bands visualized with alkaline phosphatase was normalized to ß-actin or ßIII-tubulin of the same lane. Data were plotted as units relative to the average of the control bands determined from every gel. Antibodies are listed in Table 1.

### Morphometry

For morphological analysis, immunostained neurons were reconstructed (Neurolucida system; MicroBrightField, Inc., Williston, VT, United States) by trained observers blinded to conditions and were cross-checked by a different observer who was also blinded to conditions. Neurons were classified as pyramidal neurons of layer (L) 2/3 with an apical dendrite that reaches L1 and as pyramidal neurons of L5/6 with an apical dendrite that ends in the middle layers. Spine density analysis was done using the Neurolucida system. All types of dendritic protrusions, including filopodia, were considered (addressed as “spines”), and dendritic segments of the second order or higher were analyzed. While the influence of glutamatergic signaling on dendritic development is well established, less is known about the activity-dependent mechanisms shaping axonal morphology, presumably due to the difficulty in obtaining faithful reconstructions of the axonal complexity. Slice cultures prepared from the perinatal cortex offer the advantage that interneurons differentiate entirely in vitro, and sparse transfection permits addressing the complete 3D pattern in one slice, avoiding potential errors. The neocortical interneuron types become distinguishable by morphological features around 10 days after birth in vivo and similarly in organotypic cultures. We strictly employed the morphological criteria demonstrated qualitatively in recent work focusing on local and horizontally projecting basket cells and vertically projecting translaminar non-basket cells with bitufted and arcade-shaped axons. For instance, basket cells typically feature thick main axons and delicate collaterals dotted with irregular-sized boutons and terminal elements contacting somata, as demonstrated (Gonda et al. 2023b). As in the previous work, we excluded Martinotti cells due to their highly variable extent in L1, bipolar cells due to their rather diffuse axon plexus, and neurogliaform neurons with their extremely local small-diameter plexus in middle layers (Gasterstädt et al. 2022; Gonda et al. 2023b). For AcD cells, the distance between the soma and the point of origin of the axon (axon hillock) was determined. The rarely occurring “shared root” configuration (Wahle et al. 2022) has been considered as an axon of somatic origin. The number of culture batches and neurons assessed (963 cells in total) is given in Table 2. For the assessment of presynaptic boutons of basket cells, photomicrographs of axon collaterals within the cell’s dendritic field were taken at 1000x magnification with a CCD camera mounted to a Zeiss Axiophot microscope equipped with differential interference optics. Neurons selected resided solitarily and had, in particular, no axons from other basket cells intermingled with their plexus. At first, the assessment was done with the axonally reconstructed basket cells at DIV 10. We randomly selected for each condition (control, CIQ, DQP) 10 neurons with somatic axons and 10 neurons with axons from dendrites. For each cell, we assessed a minimum of 300 μm axonal collateral length harboring 30 to > 100 boutons. Further, we plotted the density of boutons along a minimum of 5 randomly selected axonal collaterals (somatic axon cells only) of a minimum 300 μm length. We repeated the bouton size analysis with DIV 20 basket cells, and with different experimenters who were blinded to conditions. The area of the boutons was determined with MacBiophotonics (ImageJ).

**Table 2:** Number of independent preparations (culture batches) and number of neurons for the morphometric analyses.

Cell type	No. of batches	DIV 10 No. of neurons assessed			No. of batches	DIV 20 No. of neurons assessed		
		control	CIQ	DQP		control	CIQ	DQP
Pyramidal cells L2/3	8	58	27	47	4	40	36	36
Pyramidal cells L5/6	8	34	25	48	4	28	39	27
Basket cells	11	71	55	55	5	19	10	17
Non-Basket cells	11	96	62	59	5	20	23	14

### Calcium imaging

Cultures were sparsely transfected with the GCamP6f-Fishell-2 plasmid, a calcium sensor under control of the mouse distal-less 5/6 enhancer to enrich for expression in interneurons. Transfection was performed at DIV 7–8, followed by imaging at DIV 10–13 with a Leica SP5 confocal microscope. Cells were acutely exposed to CIQ or DQP, each at 15 μM in HEPES-buffered artificial cerebrospinal fluid. After imaging, the cells were fixed and stained against EGFP to confirm the interneuronal nature by means of smooth or sparsely spinous dendrites, lack of dendritic polarity, and locally branching axons.

### Statistical analysis

Data management, graphs, and statistical analysis were done with SigmaPlot 12.3 (Systat Software, Erkrath, Germany). Non-parametric ANOVA on ranks test with Dunn’s correction for multiple testing and/or non-parametric Mann–Whitney rank sum tests were conducted.

## Results

### Functional expression of GluN2D receptors in cortical interneurons

**Fig. 1:**
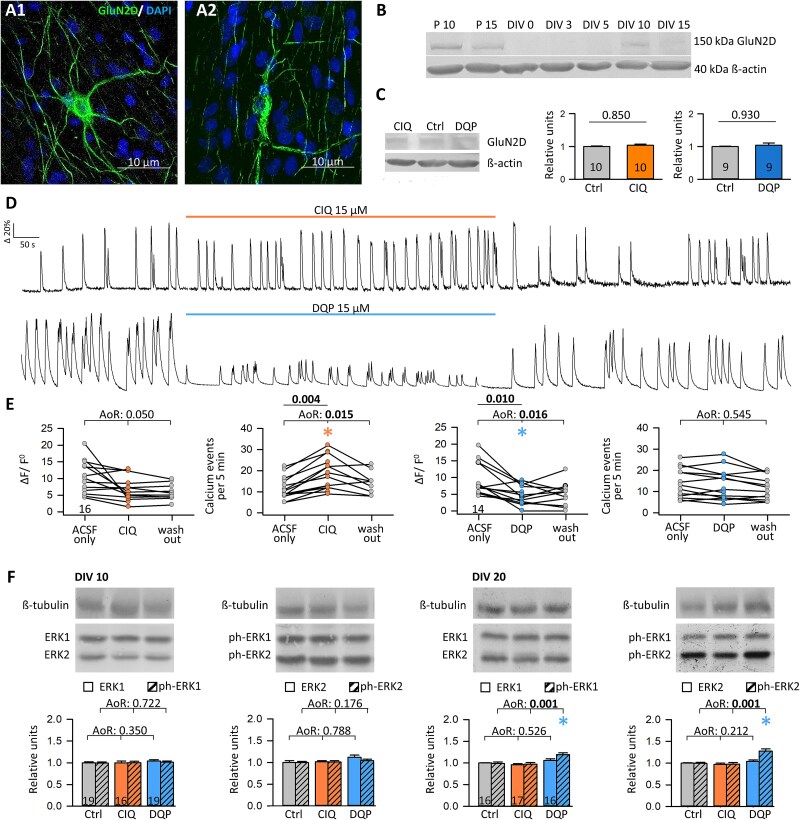
The GluN2D receptor subunit is functionally expressed in interneurons. A1, A2) GluN2D antibody staining in visual cortex organotypic cultures at DIV 11 detects multipolar and bitufted neurons with staining in somata, dendrites, and axons, indicating presynaptic receptor expression. (B) Expression of the GluN2D subunit protein in visual cortex in vivo at postnatal day (P) 10 and P15, and in vitro at DIV 0–15. Peak expression in both conditions is P10 and DIV 10, respectively. (C) Representative blot pictures of GluN2D with β-actin of the same lanes. Expression of GluN2D was not changed with CIQ or DQP treatment from DIV 5–10 (daily 1 pulse, 15 μM final concentration in the medium). Control cultures (Ctrl) received the same amount of dimethylsulfoxide in water. Plots of relative units normalized to β-actin; the average of the controls was set to 1. (D) Representative calcium traces of spontaneously active neurons in artificial cerebrospinal fluid (ACSF) before, during, and after application of CIQ or DQP, recorded at DIV 10–12. (E) Amplitude and frequency of calcium events recorded in acutely CIQ-treated (left) or DQP-treated (right) interneurons. (F) Representative blot pictures of total ERK1 and ERK2 in comparison with tyrosine phosphorylated ERK1 and ERK2 normalized to ßIII-tubulin at DIV 10 and DIV 20. The number of analyzed cultures (C, F) and recorded cells (E) is given within the graphs. ANOVA on Ranks (AoR) versus control (C, F); p-value given above the boxplots. Significant p-values in bold, colored asterisks indicate the significant differences. Mann–Whitney Rank Sum Test of ACSF only versus CIQ or DQP (E). In this and all following figures, control is shown in gray, CIQ treatment in orange, and DQP treatment in blue.

In cultures at DIV 10, GluN2D-like immunoreactivity was in somata, dendrites, and axons of bitufted and multipolar neurons (Fig. 1A). Axonal staining suggested the presence of GluN2D-containing receptors in presynapses, and thus the possibility of direct actions of the modulators at the level of the axon. Presynaptic NMDA receptors exist in pyramidal cells, and for instance, the GluN1/GluN2B receptors are expressed in the presynaptic active zone (Gill et al. 2015) react to synaptic glutamate release, and facilitate spontaneous neurotransmitter release (Banerjee et al. 2016). In the subthalamic nucleus, unmyelinated axons contain GluN2D receptors (Swanger et al. 2015), and developing interneurons of the molecular layer have presynaptic NMDA receptors, and their activation increases calcium release from ryanodine-sensitive stores, which induces neurotransmitter release (Rossi et al. 2012).

Lysates of rat visual cortex in vivo at P10 and P15 (loading 30 μg protein per lane) were blotted together with lysates from organotypic cultures (1 per lane). A single band at about 150 kDa (mass of 143.7 kDa for human GRIN2D protein according to UniProt) was detected in lysates from cortex in vivo and in vitro at DIV 10, with a decline towards DIV 15 (Fig. 1B). This matches the mRNA peak expression at P7–9 (Monyer et al. 1994), whereas the protein may peak somewhat later and remain at higher levels for some time. Moreover, the GluN2D expression did not change under chronic treatment, suggesting no homeostatic or ligand-induced scaling of subunit expression (Fig. 1C). Organotypic roller cultures become spontaneously active until DIV 10 with fast-spiking basket cells and regular-spiking non-basket cells (Klostermann and Wahle 1999). To test for functional GluN2D-containing NMDA receptors, spontaneously occurring calcium events were recorded in DIV 10–14 neurons, followed by immunofluorescence staining against the GFP domain of the construct to confirm the interneuronal nature (Fig. 1D). In basket cells and non-basket cells, the calcium event frequency significantly increased with wash-in of CIQ, while the amplitude remained unchanged. With wash-in of DQP, the calcium event amplitude significantly decreased while the calcium event frequency remained unchanged (Fig. 1E). Separating the recorded interneurons by axon origin revealed that the calcium event amplitude and event frequency measured in the soma were similar in cells with somatic axons and axons originating from dendrites although the AcD cells were presumably too few to detect subtle differences, and not all recorded cells could have been retrieved (Supplemental Fig. S1). Together, this suggested a functional expression of endogenously occurring GluN2D-containing NMDA receptors subunit in non-pyramidal cells in organotypic cortex cultures.

Inhibiting interneuronal GluN2D-containing receptors would reduce the level of inhibition, and consequently, excitatory pyramidal cells become disinhibited, and network activity could increase. To test this possibility, organotypic cultures at DIV 10 and DIV 20 were exposed acutely for 30 min to CIQ or DQP. Total ERK1 and ERK2 proteins remained at the control level at DIV 10 and DIV 20. Tyrosine phosphorylation of p42/44 ERK1/2 was increased at DIV 20 with DQP treatment (Fig. 1 F). This suggested that inhibiting interneuronal GluN2D receptors rapidly dampens the level of inhibition in favor of a higher level of network excitation, leading to enhanced signaling via the MAP kinase cascade.

### DQP treatment influences pyramidal cell development

**Fig. 2:**
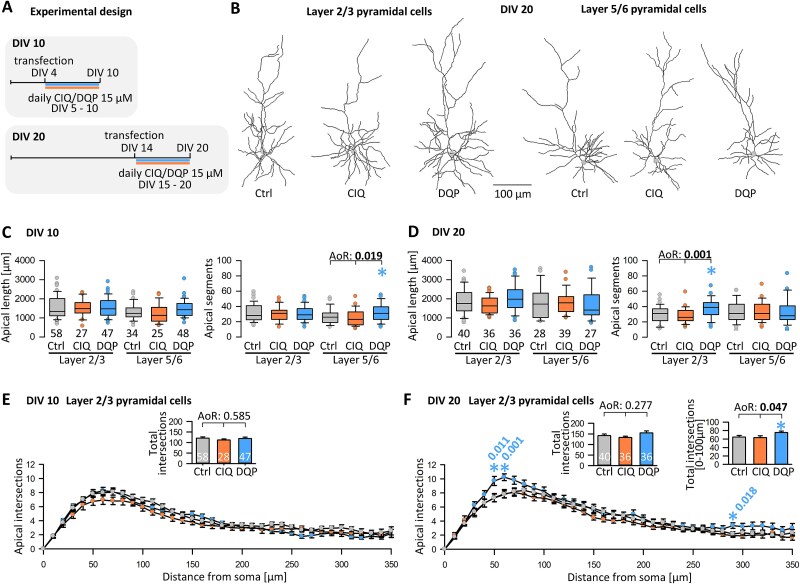
Antagonizing GluN2D-containing receptors enhances pyramidal cell dendritic growth. (A) Experimental design with treatment until DIV 10 and DIV 20. (B) Representative skeletal drawings of L2/3 and L5/6 pyramidal cells at DIV 20 of control (Ctrl), CIQ- and DQP-treated cultures. (C) Analysis of L2/3 and L5/6 pyramidal cells at DIV 10 with apical dendritic length and apical dendritic segments. Basal dendritic length and segments are reported in Supplementary Table S1. The number of neurons is given above the box plots on the left. (D) Analysis of L2/3 and L5/6 pyramidal cells at DIV 20 with apical dendritic length, apical dendritic segments, basal dendritic length, and basal dendritic segments (the average per neuron is plotted). The number of neurons is given above the box plots on the left. Note that in DQP-treated organotypic cultures, L5/6 pyramidal cells had more apical segments at DIV 10, and L2/3 pyramidal cells had more apical segments at DIV 20. (E) Sholl analysis of L2/3 pyramidal cell apical dendrites at DIV 10 reveals no differences. (F) Sholl analysis of L2/3 pyramidal cell apical dendrites at DIV 20 reveals significantly more branched dendrites within 50–100 μm from the soma and also a trend to more branches in the apical tuft ~ 300 μm. E, F) insets: The total intersections were not different due to the regional effect on branching in just a few proximal bins. ANOVA on Ranks (AoR) versus control; p-value given above the box plots. Significant p-values in bold, colored asterisks indicate the significant differences.

**Fig. 3:**
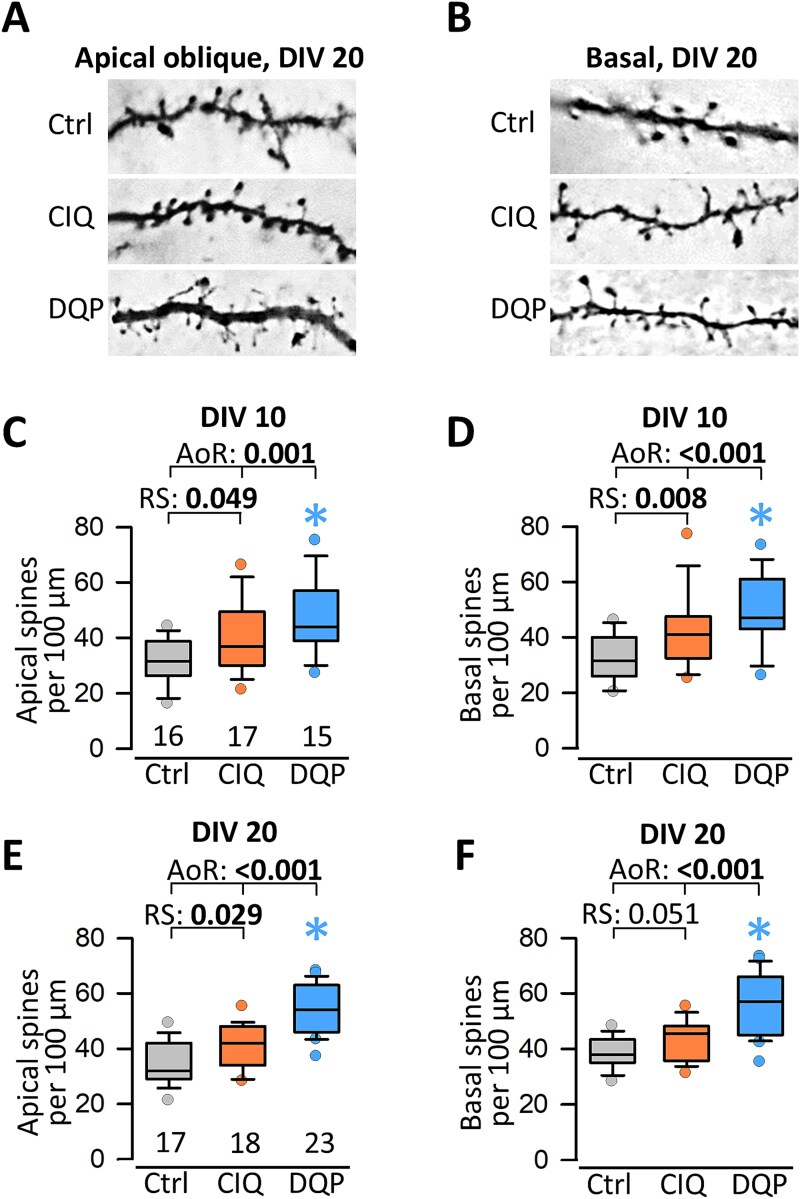
Antagonizing GluN2D-containing receptors enhances pyramidal cell dendritic spine density. (A) Representative segments of apical oblique and B. Basal dendrites segments of control (Ctrl) and CIQ- and DQP-treated pyramidal cells at DIV 20. C-F) Apical and basal spine densities at DIV 10 and DIV 20 of control, CIQ- and DQP-treated pyramidal cells. All protrusions, including filopodia, were considered. ANOVA on Ranks (AoR) versus control; p-value given above the boxplots. Pairwise testing of CIQ versus control was done with the Mann–Whitney rank sum test (RS). Significant p-values in bold, colored asterisks indicate the significant differences.

Previous work demonstrated the importance of glutamatergic signaling for the development of pyramidal cell dendrites, spines, and axonal connectivity (Hamad et al. 2011; Hamad et al. 2014; Jack et al. 2019; Gonda et al. 2020; Lauri et al. 2021). The following assessments were done at two time points with the experimental design shown in Fig. 2A. The CIQ treatment did not affect L2/3 and L5/6 pyramidal cells. In contrast, DQP treatment did affect pyramidal cells. Representative skeletal drawings of DIV 20 pyramidal neurons demonstrate more complex apical dendrites of DQP-exposed L2/3 pyramidal cells (Fig. 2B). Quantitatively, at DIV 10, the apical dendrites of L5/6 pyramidal cells had more segments, albeit not more length (Fig. 2C; Supplementary Table S1 summarizes numerical data). Matching the significantly enhanced ERK1/2 phosphorylation at DIV 20, apical dendrites of DQP-treated L2/3 pyramidal cells had more branches (Fig. 2C; Supplementary Table S1). Exemplarily, a Sholl analysis run for L2/3 pyramidal cells at DIV 10 (Fig. 2E) and DIV 20 (Fig. 2F) reported a significant increase of intersections (a measure of dendritic branching) within ~ 100 μm distance from the soma at DIV 20, and the DQP curve was overshooting the control and CIQ curves also in the apical tuft region at 300 μm distance (Fig. 2F). The total number of intersections was not different from the control at DIV 10 (inset in Fig. 2E). The insets in Fig. 2F reveal that the total intersections were not different, but the branching was significantly enhanced in DQP-treated pyramidal cells within the 50–80 μm bins close to the soma. This is the domain where the typical “apical oblique” side branches form. Basal dendrites were not affected by CIQ or DQP treatment (Supplementary Table S1). The development of dendritic spines is a highly sensitive morphological readout for even subtle alterations of network activity because spine building and maintenance are regulated by neuronal activity, in particular via ionotropic glutamate receptor signaling, and the MAP kinase pathway (Thomas and Huganir 2004; Dijkhuizen and Ghosh 2005; Wang et al. 2007). After DQP treatment at DIV 20, the density of spines on apical oblique and basal dendrites was significantly increased (Fig. 3A, B, E, F). This was in line with the results of the ERK1/2 phosphorylation. Surprisingly, spine densities were already significantly higher at DIV 10 in the absence of measurable alterations of ERK1/2 signaling (Fig. 3C, D). A certain upshift of boxes and medians was also seen with CIQ treatment, although it did not survive the ANOVA on Ranks versus control. However, Mann–Whitney rank sum tests of CIQ versus control clearly revealed an increased spine density. Together, this suggested a clear-cut growth-promoting effect for pyramidal cells via an enhanced network activity caused by DQP-evoked disinhibition and, unexpectedly, suggested that the CIQ treatment may also evoke a dysbalanced activity.

Pyramidal cell axonal development in vitro matches in vivo development and is dependent on glutamatergic signaling (Uesaka et al. 2005). For instance, pyramidal cells expressing the inhibitory hM4Di DREADD receptor display reduced activity when exposed to the ligand clozapine N-oxide, and the number of collaterals arising from the primary axon is reduced, as is the number of bouton terminaux, which are bona fide presynapses (Gasterstädt et al. 2022). To test if modulating GluN2D-containing receptors could influence axonal collateralization, we reconstructed axons of randomly selected L2/3 pyramidal cells in cultures from 2 preparations (Supplementary Fig. S2A). We observed a small developmental increase of collateral number from DIV 10–20 (Supplementary Fig. S2B, C), and a moderate increase in the density of bouton terminaux (Supplementary Fig. S2D, E), both of which explainable by ongoing maturation. However, neither CIQ nor DQP treatment altered these axonal parameters.

### Effects of CIQ and DQP treatment on interneuronal dendrites

Spontaneous glutamatergic activity can increase the length and branching of interneuronal dendrites (Klostermann and Wahle 1999; Chattopadhyaya et al. 2007; Hamad et al. 2011; Jack et al. 2019). The local density of the axonal plexus could reflect the cell’s history of activity. For instance, transfection of channelrhodopsin-YFP and repetitive DIV 10–15 optogenetic stimulation @0.5 Hz increases the local axonal complexity of basket cells but not of non-basket cells (Gonda et al. 2023b). A detailed analysis has revealed that the point of origin of the axon is important. Basket cell axons originating from axon-carrying dendrites (AcDs) grow locally denser plexuses in spontaneously active organotypic cultures; this does not require additional optogenetic stimulation. The latter promotes the local branching of basket cell axons originating from somata. In human and carnivore cortex in vivo, about 25% of the parvalbuminergic basket cells and chandelier cells, and up to 50% of the somatostatin-positive non-basket cells have AcDs (Wahle et al. 2022). In mouse, about half of the CA1 pyramidal cells display axons from basal dendrites (Thome et al. 2014). Also, in rat slice cultures, a substantial proportion of basket cells and non-basket cells features AcDs (Höfflin et al. 2017). In basket cells, these AcDs are complex and often among the longest dendrites of the cell indicating a mutual growth-promoting relationship between an AcD and the axon it carries (Gonda et al. 2023b). This prompted a detailed analysis of basket cell and non-basket cell dendrites and axons in CIQ- and DQP-treated cultures at DIV 10, at the peak of the endogenous GluN2D expression.

**Fig. 4:**
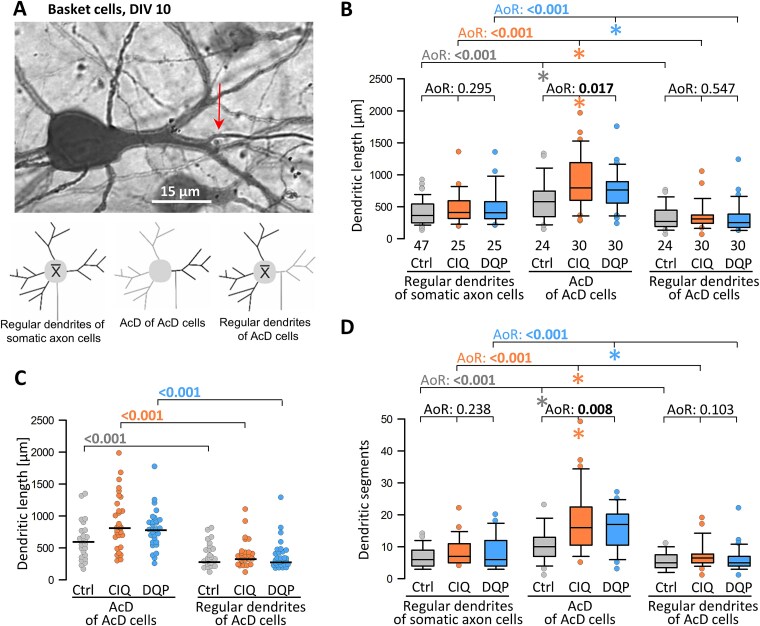
The influence of axon origin and GluN2D signaling on basket cell dendritic development. (A) Representative basket cell with an axon from a secondary dendrite. Small sketches help to identify regular dendrites and AcDs. (B) Comparison of the mean dendritic length of somatic axon cells with regular dendrites and AcD cells with their AcD (single value) and their regular dendrites (average per cell). (C) Comparison of dendritic length within AcD cells with the AcD (single value) and the regular dendrites (average per cell) demonstrated as a dot density plot to better reveal the variability and to run pairwise tests. (D) Comparison of mean dendritic segments of basket cells with somatic axons with regular dendrites and AcD cells with their AcD (single value) and their regular dendrites (average per cell). A, B, D) ANOVA on ranks (AoR) versus control; p-value given above the box plots. Significant p-values in bold, colored asterisks indicate the significant differences. (C) Mann–Whitney rank sum test; p-values are given. Significant differences in bold. The n is given in the graphs.

An AcD basket cell is shown in Fig. 4A. Regular dendrites of basket cells were not affected by CIQ or DQP treatment, neither at DIV 10 (Fig. 4B-D) nor at DIV 20 (Supplementary Fig. S3A, B). Only the detailed comparison revealed that the AcD (small sketches in Fig. 4A) of the untreated control basket cells was, on average, a more complex dendrite, which was longer and more branched than the regular dendrites of these AcD cells and more complex than the dendrites of basket cells with somatic axons. The AcD of CIQ-treated basket cells was even longer and more branched, suggesting a growth-promoting action of GluN2D receptor activation for the AcD of basket cells. Unexpectedly, the AcD of DQP-treated basket cells was also longer and more branched (Fig. 4B, C, D), presumably via enhanced network activity. These results confirm our previous data obtained at DIV 15 (Gonda et al. 2023b), now with an independent data set at DIV 10. It suggested that despite the presumably age-related higher morphological variance, the AcD of basket cells already starts to enlarge around DIV 10.

**Fig. 5:**
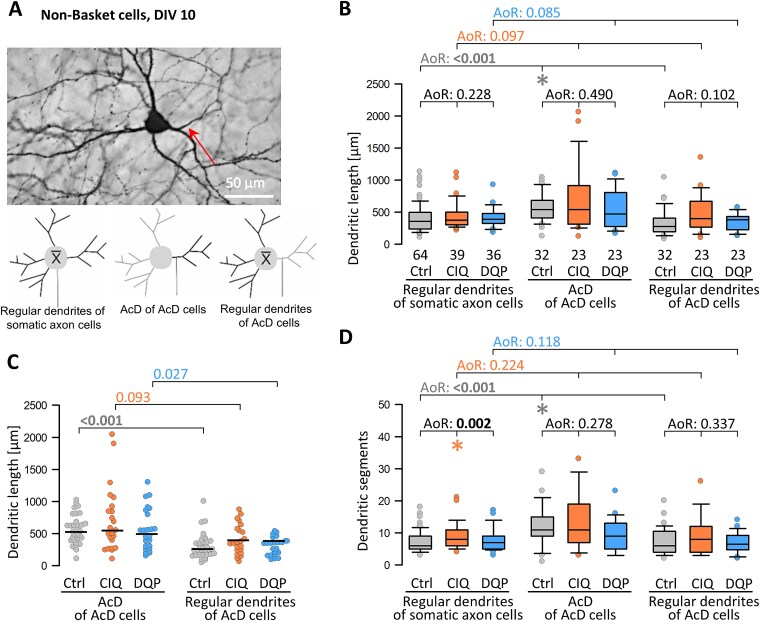
The influence of axon origin and GluN2D signaling on non-basket cell dendritic development. (A) Representative non-basket cell with an axon from a primary dendrite. Small sketches help to identify regular dendrites and AcDs. (B) Comparison of the mean dendritic length of somatic axon cells with regular dendrites and AcD cells with their AcD (single value) and their regular dendrites (average per cell). (C) Comparison of dendritic length within AcD cells with the AcD (single value) and the regular dendrites (average per cell) demonstrated as a dot density plot to better reveal the variability and to run pairwise tests. (D) Comparison of mean dendritic segments of somatic axon cells with just regular dendrites and AcD cells with their AcD (single value) and their regular dendrites (average per cell). A, B, D) ANOVA on ranks (AoR) versus control; p-value given above the box plots. Significant p-values in bold, colored asterisks indicate the significant differences. (C) Mann–Whitney rank sum test; p-values are given. Significant differences in bold. The n is given in the graphs.

Non-basket cells barely respond to DIV 10–15 optogenetic stimulation; however, the AcD is more complex than the remaining dendrites of the AcD cells at DIV 15 (Gonda et al. 2023b). An AcD non-basket cell is shown in Fig. 5A. CIQ and DQP treatment did not affect the regular dendrites of non-basket cells at DIV 10 (Fig. 5B-D) nor at DIV 20 (Supplementary Fig. S3C, D). At DIV 10, in control non-basket cells, the AcDs were longer and more branched than regular dendrites of non-basket cells with somatic axons. CIQ-treated non-basket cells with somatic axons had more branches than control cells, but there was no effect on length. The AcDs of CIQ- and DQP-treated non-basket cells were not significantly different from their regular dendrites nor from the dendrites of non-basket cells with somatic axons (Fig. 5B-D), although some CIQ-treated cells had quite large dimensions. Together, except for a rather moderate growth-promoting effect of CIQ on regular dendrites, the non-basket cells did not respond with dendritic growth to the treatments.

### Effects of CIQ and DQP-1105 treatment on basket cell axon development

For the axons, we determined total axonal length, the number of axonal nodes (branch points) per 1000 μm the number of bouton terminaux per 1000 μm, the terminal segment length, the proportion of growth cones in percent from all endings, and the z-span of the plexus because roller-type organotypic cultures undergo a flattening over time. The number of axons shown in the Figures is lower than the number of cells assessed for dendritic parameters because not all cells had completely stained axon plexuses.

**Fig. 6:**
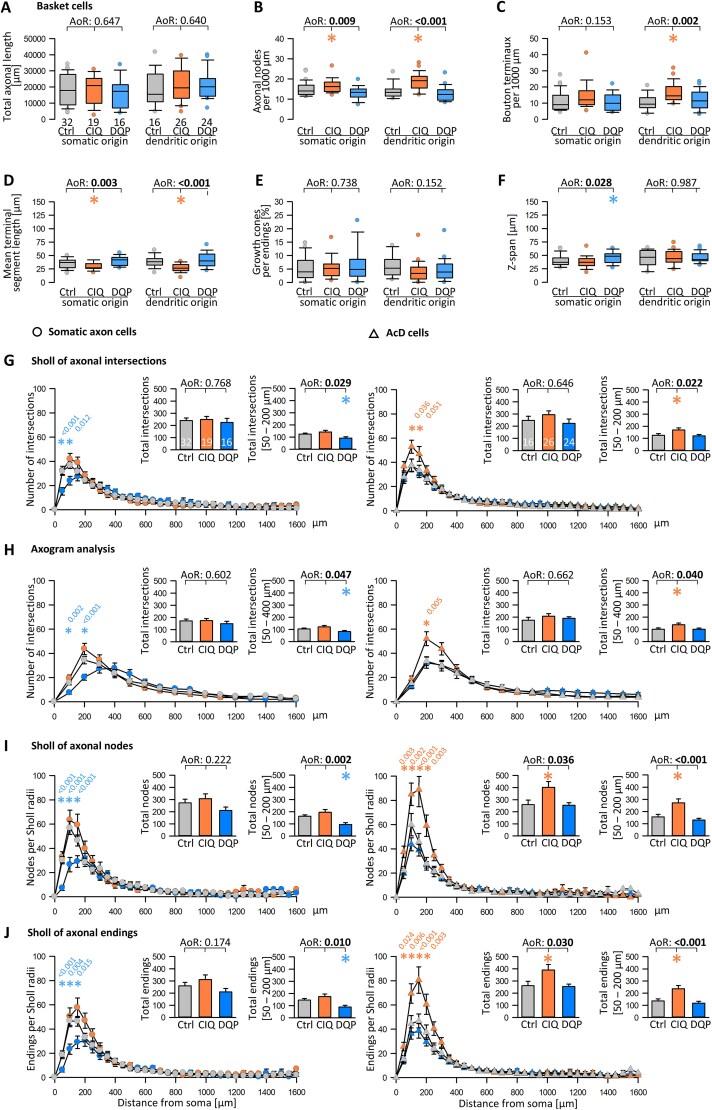
GluN2D signaling influences the development of basket cell axons. A-F) Parameters of basket cell axons with somatic and dendritic origin. (A) Total axonal length. (B) Number of axonal branch points (nodes) per 1000 μm. (C) Number of Bouton terminaux per 1000 μm. (D) Mean length of the terminal segments, average per cell, is plotted. (E) Proportion of axonal endings tipped with growth cones. (F) The z-span (depth) of the axon plexus, the maximal depth value is plotted per cell. G-J) Sholl-type analyses of axons with origin from somata (left column; circles) and AcD (right column; triangles). Axon values from the start to 1600 μm distance were considered (arbitrary cut-off, only a few axons reached beyond that distance). (G) Classical soma-centered Sholl of axonal intersections with 50 μm circle distance. (H) Linear axogram analysis with 100 μm bins starting at the origin of the axon. I) The number of nodes per 50 μm radius. (J) The number of endings per 50 μm radius. The small insets report the total intersections, nodes, endings, and the intersections, nodes, endings within the few bins where the ANOVA detected significant differences. ANOVA on ranks (AoR) versus control; p-value given above the box plots. Significant p-values in bold, colored p-values indicate the significant differences of the CIQ (orange) or DQP (blue) treatment versus control; colored asterisks indicate the significant differences in the box plots and bar graphs.

Total basket cell axonal length was not altered by CIQ or DQP treatment, nor by axon origin (Fig. 6A). However, with CIQ treatment, basket cell axons of somatic and dendritic origin had more nodes (Fig. 6B). Basket cell axons originating from a dendrite had more bouton terminaux, which we determined as a proxy for presynapse formation (Fig. 6C). Basket cell axons of somatic and of dendritic origin had a shorter mean length of the terminal segments (Fig. 6D), which are the short rows of varicosities in close apposition to pyramidal cell somata and proximal dendrites. This suggested that activating GluN2D receptors helps to mature the terminal elements, possibly with a contribution of GABA released by these terminals (Chattopadhyaya et al. 2007). With CIQ, the terminal elements were in the length range measured in DIV 15 basket cells after optogenetic depolarization (Gonda et al. 2023b), which also implicates GABA release as a major mediator for axonal branching and terminal maturation, although the axonal boutons along basket cell axon collaterals have remained smaller in size. The proportion of axon endings with growth cones varied from zero to > 25% from cell to cell (Fig. 6E), irrespective of treatment. The z-span of the basket cell plexuses sampled from the CIQ-treated cultures tended to be a bit smaller than in control and DQP-treated cultures (Fig. 6F), which would rather work against us because a narrow plexus usually means less tissue depth and, thus, less 3D space for local branching.

Next, we ran Sholl analyses (Fig. 6G-J) for axons of somatic origin (left, circles) and axons of dendritic origin (right, triangles). We used the classical soma-centered Sholl of axonal intersections (50 μm circle distance) (Fig. 6G), a linear axogram analysis with 100 μm bins starting at the origin of the axon (Fig. 6H), the number of nodes per 50 μm radius (Fig. 6I), and the number of endings per 50 μm radius (Fig. 6J). For basket cells with somatic axons, the curve of the DQP-treated cells was systematically undershooting the curve of the control cells in the ~ 200 μm radius around the soma within the parent cell’s dendritic field. The curve of the CIQ-treated basket cells tended to overshoot the controls in the same region, albeit not yet significantly. In contrast, for basket cells with axons from a dendrite (Fig. 6G-J in the column to the right), the curve of the DQP-treated cells was close to the control curve. Strikingly, the curve of the CIQ-treated basket cells was substantially above the control curve in a ~ 300 μm radius around the soma, being significant in all four parameters (Fig. 6G-J in the column to the right). The small insets show that total nodes and endings were significantly increased, and intersections were significantly increased in the bins proximal to the somata. This suggested that first, activating GluN2D-containing receptors accelerates the local branching of basket cell axons. Second, a growth-promoting effect mediated by the AcD can neutralize the inhibitory effect of DQP. Together, we have identified a novel mechanism of basket cell axonal growth.

### Effects of CIQ and DQP-1105 treatment on non-basket cell axon development

**Fig. 7:**
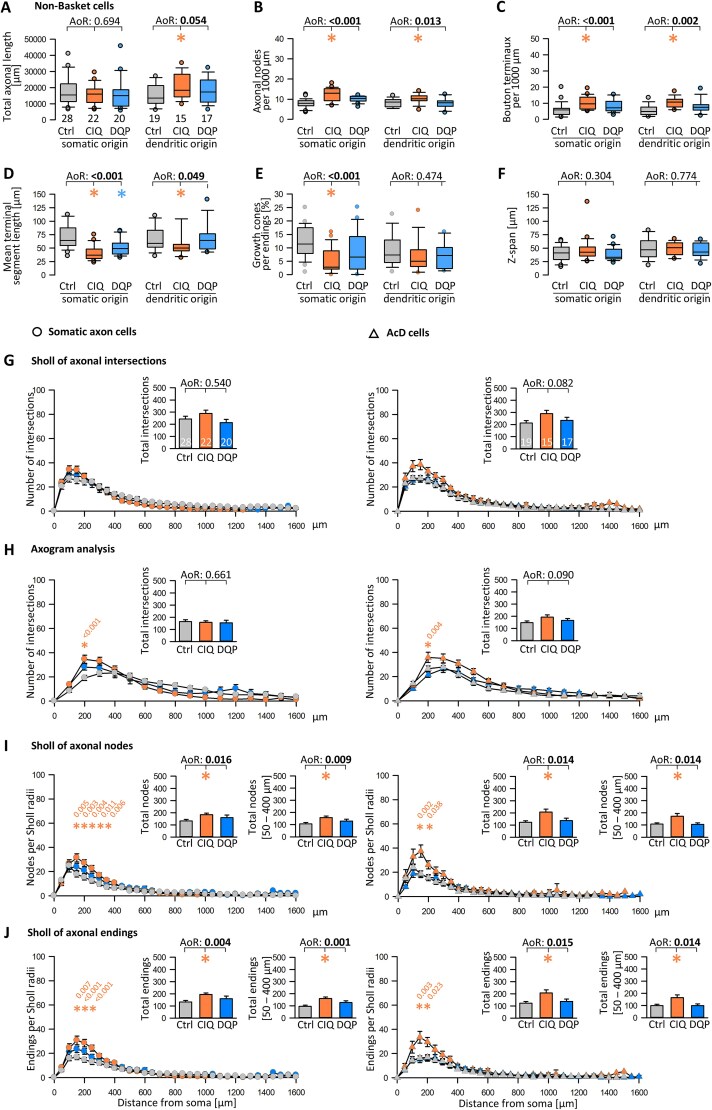
GluN2D signaling influences the development of non-basket cell axons. A-F) Parameters of non-basket cell axons with somatic and dendritic origin. (A) Total axonal length. (B) Number of axonal branch points (nodes) per 1000 μm. (C) Number of Bouton terminaux per 1000 μm. (D) The mean length of the terminal segments, average per cell, is plotted. (E) Proportion of axonal endings tipped with growth cones. (F) The z-span (depth) of the axon plexus, the maximal depth value is plotted per cell. G-J) Sholl-type analyses of axons with origin from somata (left column, circles) and AcD (right column, triangles). Axon values from the start to 1600 μm distance were considered (arbitrary cut-off, only a few axons reached beyond that distance). (G) Classical soma-centered Sholl of axonal intersections with 50 μm circle distance. (H) Linear axogram analysis with 100 μm bins starting at the origin of the axon. I) The number of nodes per 50 μm radius. (J) The number of endings per 50 μm radius. The small insets report the total intersections, nodes, endings, and the intersections, nodes, endings within the few bins where the ANOVA detected significant differences. ANOVA on Ranks (AoR) versus control; p-value given above the box plots. Significant p-values in bold, colored p-values indicate the significant differences of the CIQ or DQP treatment versus control; colored asterisks indicate the significant differences in the box plots and bar graphs.

The total axonal length of non-basket cell axons was not influenced by CIQ or DQP (Fig. 7A). The number of nodes and bouton terminaux per 1000 μm was increased by CIQ treatment (Fig. 7B, C). The terminal segment length was shorter in CIQ and in DQP-treated cells (Fig. 7D), albeit remaining longer than the basket cell axon terminals’ average length, and non-basket cells have longer terminal elements (Gonda et al. 2023b). Again, this was observed in cells with somatic axons and AcD cells. It is suggested that the depolarizing action of CIQ has elicited the effects. Surprisingly, with CIQ treatment, the proportion of growth cones was significantly reduced in axons emerging from somata but not in axons from dendrites (Fig. 7E). The z-span of the non-basket cell axon plexuses did not vary between conditions (Fig. 7F). The Sholl assessment revealed that the curve of the CIQ-treated non-basket cell axons was always overshooting the control cell curve of the somatic axon cells (Fig. 7G-J in the column to the left; circles) and of the AcD cells (Fig. 7G-J in the column to the right, triangles). Significant differences were consistently seen in proximal bins up to ~ 300 μm from the soma in the axogram analysis, the Sholl analysis of axonal nodes (Fig. 7I), and axonal endings (Fig. 7J). The insets reporting total intersections and intersections in the 50–400 μm bins (Fig. 7I, J) confirm the growth-promoting action of CIQ. Non-basket cell axons from an AcD were shifted up within the same bins and to about the same degree as axons of somatic origin, suggesting no additional growth effect of the AcD configuration. Overall, the peak values of nodes and endings within the dendritic field were clearly lower than those of basket cells, an argument for the reliability of our classification. Our previous study revealed no remarkable AcD-mediated growth effect for non-basket cells (Gonda et al. 2023b), although up to 50% are AcD cells (Höfflin et al. 2017; Wahle et al. 2022). The present results now suggest that activating non-basket cells with CIQ increased axonal complexity in early postnatal development and that the AcD configuration had no additive effect.

Overall, the proportion of growth cones varied substantially between the interneurons from zero to up to > 25% of all endings (Fig. 6E; Fig. 7E). One might expect neurons with short axons to sport more endings with growth cones. Testing for an association between axonal length and the proportion of growth cones yielded an extremely inconsistent picture and overall low R^2^ values, suggesting no correlation. Axonal growth is a highly dynamic “grow-pause-retract-grow” process, and in cortical projection neurons, growth cone pausing is closely related to axonal branching via frequency-dependent calcium transients that differ from branch to branch. Thus, every individual axon and even every branch is elongating or pausing at its own pace, and higher calcium transient frequencies correlate with more rapid growth (Kalil et al. 2011). Yet, the intracellular mechanisms in interneurons must differ from those described for projection neurons because, for instance, the latter involve CamKII as a central signal integrator (Kalil et al. 2011). Yet, CamKII is not expressed in cortical interneurons. Moreover, activity can also exert a “stop” function. For instance, optogenetically stimulated infragranular pyramidal cells form shorter apical dendrites (Gonda et al. 2023a), generating the typical apically stunted morphology of L5/6 callosal and corticothalamic projection neurons. Here, we observed that CIQ significantly reduced the proportion of growth cones of non-basket cell axons emerging from somata but not significantly of axons from dendrites, and terminal segment length was reduced in both axons of somatic and dendritic origin. This did not come with a reduction of total axon length, which one could expect would be as simple as an activity-dependent growth cone collapse. Rather, non-basket cell axons from dendrites tended to be longer. If total length is maintained or even longer despite impaired tip growth and shorter terminal elements, interstitial growth must have been promoted by CIQ. Indeed, the number of nodes was significantly higher in axons of somatic and dendritic origin (Fig. 7B).

Next, we tested if the length of the AcD is associated with the distance between the point of axon origin and the parent somata. In CIQ- and DQP-treated basket cells, the axons emerged at significantly larger distances to the soma (Supplementary Fig. S4A). In non-basket cells, the distances were not different from control (Supplementary Fig. S4B), presumably because the AcDs of non-basket cells were not substantially longer than regular dendrites. Together, activating GluN2D-containing receptors in basket cells and non-basket cells leads to more complex axons, and the AcD configuration adds to it either by a direct growth-promoting or growth-permissive action.

### Effects of CIQ and DQP-1105 treatment on interneuronal presynapse structure and neurochemistry

**Fig. 8:**
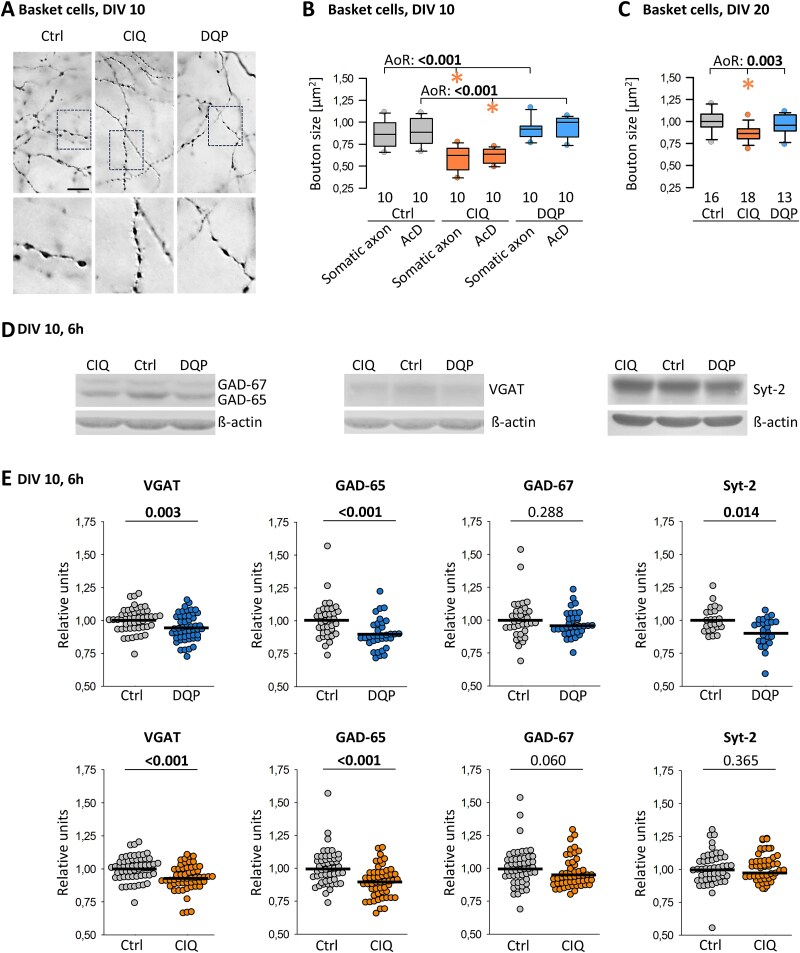
Effects of CIQ and DQP treatment on interneuronal presynapse structure and neurochemistry. (A) Boutons of basket cell axons at DIV 10. (B) Bouton size of CIQ-treated basket cells is reduced in axons of somatic and dendritic (AcD) origin at DIV 10. (C) Bouton size of CIQ-treated basket cells is reduced at DIV 20. (D) Representative protein blots for GAD-65/67, VGAT, and Syt-2. Cultures had been treated with CIQ and DQP from DIV 5–10, and lysates were prepared 6 h after the last pulse of CIQ or DQP. (E) Quantification of VGAT, GAD-65, GAD-67, and Syt-2 expression in cultures treated with DQP (upper row) and with CIQ (lower row). Note that control values are in part the same for the two treatments. Every dot is the value of one culture, plotted in relative units normalized to β-actin; gel by gel, the average of the controls has been set to 1. The mean is indicated by the line. Mann–Whitney rank sum test versus control, p-value given above the plots, significant p-values in bold. The number of lysates is reported in Supplementary Table S2.

An established criterion of basket cells is irregular-sized boutons along third and higher-order collaterals and the perisomatic terminal elements. Visual inspection of CIQ-treated basket cells at DIV 10 gave the impression that these boutons appear more delicate than in control or DQP-treated cultures (Fig. 8A). Therefore, bouton area was determined from high power photomicrographs of axon collaterals at various z-levels within the parent cell dendritic field strictly paying attention to measure only boutons exactly in the focal plane. For each condition, 20 cells were randomly selected, 10 with somatic axons and 10 with an axon from a dendrite. The hypothesis was that basket cells with an AcD allocate more resources to collateralization and less to the formation of presynapses. The bouton density was determined at DIV 10 and was found to be similar (on average 22.8 boutons/100 μm axon collateral in control, 24.5 boutons/100 μm in CIQ-treated cells, and 24.6 boutons/100 μm in DQP-treated cells). CIQ-treated basket cell axons had significantly smaller boutons. This was the case for axons with somatic as well as with dendritic origin (Fig. 8B) which argued for an effect of the CIQ treatment. A second set of basket cells (axons not reconstructed, cells not separated by axon origin) randomly selected in DIV 20 cultures was analyzed by different experimenters. CIQ treatment from DIV 15–20 also resulted in significantly smaller boutons (Fig. 8C). This suggested that activating GluN2D receptors on basket cells affects presynapse development.

The rapid activation of ERK1/2 upon acute treatment, as well as the CIQ effect on basket cell presynapses, prompted an analysis of selected proteins known to respond to activity during development, to be involved in interneuron function, or dendritic spine formation. For instance, glutamic acid decarboxylase isoforms 65 and 67 (GAD-65/67) and GABA are regulated by activity, influence basket cell axon bouton size and density, and GAD misregulation can indicate pathological activity (Patz et al. 2003; Chattopadhyaya et al. 2007). The calcium sensor synaptotagmin-2 (Syt-2) is important for the fast-spiking phenotype of basket cells (Sommeijer and Levelt 2012), is located in presynapses, as is the vesicular GABA transporter VGAT, and is sensitive to activity (Engelhardt et al. 2018). Therefore, expression was tested after DIV 5–10 treatment at DIV 10, the peak of the endogenous GluN2D expression, and after DIV 15–20 treatment at DIV 20. Detecting up to 10 proteins in a single lysate (Engelhardt et al. 2018) enabled the assessment of individual proteins in up to 60 lysates.

We hypothesized that stimulating interneurons via GluN2D-containing receptors would increase protein expression, whereas inhibiting the receptors would decrease expression. Most pre- and postsynaptic proteins assessed (GluN2A, p70/synapsin-1, p38/synaptophysin, PSD-95, GABA_A_Rα1) were not altered by the treatments and thus served as perfect internal control for those proteins that did change (Supplementary Table S2). Also, GFAP was not altered, although astrocytes express GluN2C-containing receptors (Alsaad et al. 2019), which might become activated by CIQ, potentially leading to higher GFAP expression as reported under pathological conditions (Sofroniew and Vinters 2010) (Supplementary Table S2). At DIV 10, CIQ treatment indeed upregulated interneuron-specific Kv3.1b, which is mediating the fast-spiking phenotype, and which depends in expression on activity and neurotrophic factors (Grabert and Wahle 2008). However, the effect was short-lived and only seen after 3 h of exposure. Rather, it turned out that several interneuron-enriched proteins were reduced, and that longer exposure time to the modulators is required to detect effects. At DIV 10, after ~ 6 h of exposure to DQP, the expression of GluN2B, and the presynaptic interneuronal proteins Syt-2, GAD-65, and VGAT was significantly reduced. Unexpectedly, 6 h of exposure to CIQ also reduced GAD-65 and VGAT (Fig. 8D, E). Further, at DIV 20, Syt-2 and GAD-65 were reduced by DQP treatment, and GAD-65 was reduced by CIQ treatment. Remarkably, GAD-67 expression remained unchanged (Supplementary Table S2). The absence of effect after 3 h versus effects seen at 6 h suggested that every daily pulse of the compounds has elicited such a transient reduction, with levels normalizing over the following hours until the next pulse. Together, the results are evidence for a substantial albeit transient misregulation of presynaptic interneuronal markers by inhibiting as well as by activating GluN2D-containing receptors.

## Discussion

The results confirm the growth-promoting role of the AcD configuration, in particular for basket cells as early as DIV 10, and it seems a mutual benefit for the dendrite and for the axon it carries. Moreover, the results suggest a role of GluN2D receptor signaling for axonal maturation of cortical interneurons. AcDs are privileged dendrites with lower activation thresholds, higher intrinsic excitability, and stronger dendritic spikes such that their excitatory inputs can easily trigger action potential output (Thome et al. 2014). The morphogenetic role of the AcD configuration has been almost accidentally found when testing if optogenetically evoked depolarizations could trigger neurite growth of pyramidal cells (Gonda et al. 2023a) and basket cells and non-basket cells (Gonda et al. 2023b). Importantly, the growth-promoting effect has been seen already in unstimulated control basket cells, which grow more complex AcDs and axons with denser arborization within the dendritic field of the parent cell. Now, in the present study, untreated control basket cells had already grown larger AcDs already at DIV 10, and with CIQ, the AcDs had grown even larger. With CIQ stimulation, basket cells with somatic axons tended to have more axonal branches within the dendritic field, and a quite dramatic acceleration of local branching was observed for basket cell axons emerging from AcDs. A consistent growth-promoting effect of CIQ was seen for non-basket cell axons, and this effect did not depend on the axon origin. Overall, this is in line with the highly dynamic nature of inhibitory axons (Frias and Wierenga 2013). It argues against a hard-set coupling of AcD growth and axon growth. Rather, the AcD can grow, whereas the axon it carries can remain underdeveloped. Currently, we do not yet fully understand how an axon could end up on a dendrite. A simple mechanism is that it may directly sprout from a dendrite. Alternatively, a dendrite happens to sprout from a soma close to the axon hillock, and the axon is rather passively hitchhiking away from the soma. The so-called “shared root” configuration could be an intermediate. The longer distances between axon origin and parent soma observed for basket cells (independent of treatment) but not the non-basket cells (Fig. 7) might support such a scenario, at least for an early stage of development. However, the remarkable quantitative differences between cell types and mammalian species and the similarity within individuals of a given species (Thome et al. 2014; Wahle et al. 2022; Benavides et al. 2024) argue against a stochastic process. Rather, it suggests type-specific regulatory mechanisms, also because the AcD configuration has substantial consequences for the electrophysiological behavior of the neurons.

The fact that mainly the basket cell axons from AcDs respond to the CIQ stimulation suggests that the trigger is the activity of this special dendrite. With depolarization, the AcD can fire the basket cell axon, which will release GABA. During development, GABA has a morphogenetic role. Depolarizing GABA might play a role during the time window analyzed and has been reported to promote morphological maturation of pyramidal cells (Cancedda et al. 2007). However, we did not see any increased complexity of pyramidal cell dendrites after CIQ treatment, although they did form more spines. Further, GABA can directly act on inhibitory axons and regulate their development. Depressing GABA synthesis by GAD-67 knockdown in basket cells leads to decreased axonal branching, smaller presynaptic terminals, and reduced perisomatic synapse formation (Chattopadhyaya et al. 2007). Since GAD-67 protein was entirely unaffected by activating or inhibiting GluN2D-containing receptors, such a general regulation seems unlikely; rather, the deficits precipitated at the inhibitory presynapses. The smaller size of basket cell axonal boutons seen with CIQ might be a consequence of allocating material to axonal growth, but together with the reduced expression of GAD-65 and VGAT, it might also be a homeostatic response to keep CIQ-activated interneurons in check. Albeit transient, the repetitive reduction of proteins essential for interneuronal presynaptic function could have paved the way towards disinhibition. In part, the CIQ effects resemble those seen in after exposure to the pro-inflammatory leukemia inhibitory factor, which results in reduced expression of GAD-65, Syt-2, smaller basket cell axonal boutons, and pyramidall cell hyperexcitability (Engelhardt et al. 2018).

For the DQP treatment, we expected hypomorphic interneurons and underdeveloped basket cell axons. This was the case for basket cell axons of somatic origin, where the local branching remained well below the level of control basket cells with somatic axons. However, despite inhibition, DQP-treated basket cell axons emerging from AcDs had a local branching similar to that of control basket cell axons emerging from AcDs. Thus, the AcD configuration seems to act against the inhibitory action of DQP. The intracellular mechanisms remain to be unraveled and might involve the interaction of GluN2D with the tyrosine kinase c-Abl, which is tightly regulated during development and influences dendritic, axonal, and presynaptic maturation (Motaln and Rogelj 2023) or with the presynaptic CtBP1 (Tom Dieck et al. 2005). Unexpectedly, however, the DQP treatment did not result in underdeveloped interneuronal dendrites. Rather, resembling the CIQ action, DQP-treated basket cells also had longer AcDs.

The efficiency of the DQP treatment has been demonstrated by the increased ERK1/2 phosphorylation and an increase in apical dendritic branching, first of ontogenetically older infragranular pyramidal neurons at DIV 10, and DIV 20 of the ontogenetically younger supragranular pyramidal neurons. L2/3 pyramidal cells are more responsive to enhanced network activity and, presumably, the activation of glutamate receptors (Hamad et al. 2011; Hamad et al. 2014; Jack et al. 2019) and/or downstream BDNF signaling (McAllister et al. 1995; Wirth et al. 2003) could have mediated these changes. Pyramidal cell spine formation is promoted by activity in cooperation with BDNF and is regulated via ERK1/2. Indeed, pyramidal cell spine density was increased in DQP-treated cultures already at DIV 10 and DIV 20, and the reduced expression of GluN2B observed at DIV 10 might have helped to stabilize these spines. Thus, repetitively inhibiting interneuronal activity seems efficient enough to temporarily enhance network activity, which then becomes growth-promoting for pyramidal cell development. A higher availability of TrkB ligands secreted from excitatory neurons possibly has prevented the underdevelopment of dendrites of the interneurons, which intensely express TrkB receptors (Gorba and Wahle 1999). The activation of ERK1/2 after DQP treatment resembles the effect of inhibiting GABAergic transmission for 40–60 min with picrotoxin in slices of ~P30 rat cortex and dissociated cultures (Fiore et al. 1993; Yamagata et al. 2013). Moreover, a strong ERK phosphorylation has been reported in L2/3 pyramidal cells (Yamagata et al. 2013), the apical dendrites of which were growing in DQP-treated cultures.

We suggest that the enhanced network activity has promoted the growth of the AcDs in DQP-treated basket cells. Yet, the activity between DIV 5–10 was apparently still too low to also promote basket cell axon growth. At DIV 15, spontaneous activity, together with the AcD configuration, clearly accelerates basket cell local axonal branching without requiring any further stimulation (Gonda et al. 2023b). This is in line with the interpretation that the AcD and the axon it carries do not necessarily develop synchronously and that dendritic growth precedes the increase of local axonal branching. Non-basket cell axons were less affected by DQP treatment, and their local branching was similar to control cells. Also, non-basket cell axons barely respond to optogenetic depolarization at DIV 15 (Gonda et al. 2023b). However, axons of non-basket cells responded to CIQ, suggesting a role of GluN2D receptors for this class of interneurons, too.

A starting point of our analysis has been the report that DQP administered via i.p. injection at P7–9 leads at P21 to lower IPSC frequency and underdeveloped basket cell dendrites (Hanson et al. 2019). The finding seems in line with a report on impaired accessory olfactory bulb mitral cell dendritic branching in GluN2D knockout mice (Yamamoto et al. 2017). We could not confirm the data reported by Hanson et al. (2019). We can only speculate on the reasons. First, the morphological impairment has been demonstrated by only about 10 basket cells per condition (Hanson et al. 2019). Given the variability, a sampling error might have occurred. If a small control sample comprises, by chance, more cells with complex AcDs, and the DQP sample has more somatic axon cells, it may look as if DQP has impaired dendrite development. A growth-promoting effect of the AcD configuration was not yet known in 2019. Generally, with intraperitoneal administration, it remains unclear how much of a drug reaches the brain, and monocausality will be challenging to prove. Uptake and transport will necessarily be via the bloodstream, and lymphocytes express GluN2C/D (Miglio et al. 2005). The drug might then alter the neuroimmune crosstalk. In the brain, DQP will presumably target all GluN2C/2D-expressing cells. For instance, inhibiting GluN2D-containing receptors on dopamine neurons enhances their survival (Zhang et al. 2024), potentially leading to an imbalance of dopaminergic transmission, which in turn has a morphogenetic role for cortical parvalbuminergic basket cells (Porter et al. 1999; Tomasella et al. 2018). Altering local processing via striatal GluN2D-positive cholinergic interneurons and by inhibiting the GluN2D-positive subthalamic nucleus, the output of the basal ganglia motor system (Swanger et al. 2015) will be affected, and in turn, the rhythm of glutamatergic thalamocortical inputs could be altered. Thalamic axons target parvalbuminergic basket neurons to elicit feedforward inhibition, and glutamatergic signaling is essential for the maturation of parvalbuminergic neurons (Patz et al. 2004). In mice, the lack of GluN2D results in reduced motility, a depressive-like state, and stress (Yamamoto et al. 2017). The increased stress level has already been implicated in the impairment of mitral cell dendritic development (Yamamoto et al. 2017). GluN2D receptor inhibition via systemic DQP injections decreases motor activity and theta oscillations in parietal cortex (Pálfi et al. 2021). The lower activity might contribute to the underdevelopment of parvalbuminergic neurons in the sensorimotor cortex, and stress is well known to impair the structure and function of fast-spiking neurons (Perlman et al. 2021; Gibel et al. 2022).

**Fig. 9:**
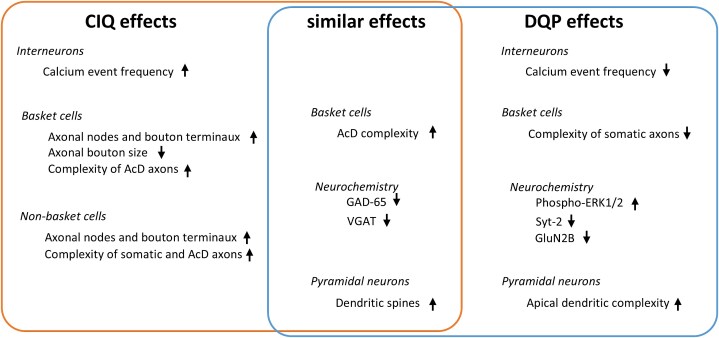
Graphical summary: Effects of CIQ and DPQ treatment on interneurons, pyramidal cells, and the neurochemistry. Arrows up, increase or accelerate development; arrows down, decrease or underdevelopment.

The most puzzling finding was that the agonist CIQ and the antagonist DQP elicited in part highly similar effects. Both caused more complex AcD in basket cells and thus, possibly indirectly, longer distances between the soma and the point of axon origin, both caused higher spine densities on pyramidal cell dendrites, both reduced the levels of GAD-65 and VGAT (see Graphical Summary Fig. 9). With DQP treatment it may be due to the disinhibition. For the CIQ treatment, we can at this moment only speculate on the underlying mechanisms. They may relate to the findings that both gain and loss of function of GluN2D receptors will result in hyperexcitability due to impaired interneuronal physiology (see Introduction).

Finally, we address several limitations. The box plots showed quite a large variability of the morphological parameters. The reason could be a heterochronous maturation, with some cells being advanced while others being less well differentiated at DIV 10. Further, the calcium imaging revealed that some respond stronger than others spontaneously at baseline and to the drugs, suggesting that individual activity levels as well as receptor expression vary between cells (see Fig. 1E). Consequently, the strength of the morphogenetic influence of CIQ and DQP likely varied between cells. Our bulk analysis sampled without preselection all cells that were completely stained and classifiable strictly by morphological criteria of the axonal pattern. This way, we could not avoid cells that were functionally too immature or not yet well enough integrated into the network at DIV 10. To cope with this, we analyzed substantial numbers of cells from many independent preparations (see Table 2), focusing on the strong significances obtained with stringent statistical tests.

## Supplementary Material

Supplementary_Materials_Kohler_et_al_2025_bhaf136
