# Supplementary material for: Activation of GluN2D-containing NMDA receptors promotes development of axons and axon-carrying dendrites of cortical interneurons
Source: Cereb Cortex. 2025 Jun 6;35(6):bhaf136. doi: 10.1093/cercor/bhaf136 (PMC12141203; doi:10.1093/cercor/bhaf136)
Supplement: Supplementary_Materials_Kohler_et_al_2025_bhaf136 [file supplementary_materials_kohler_et_al_2025_bhaf136.pdf]

Supplementary Materials

Activation of GluN2D-containing NMDA receptors promotes development of axons and axon-carrying dendrites of cortical interneurons.

Ina Köhler[1§]*, Lisa-Marie Rennau[1§], Leon Hoffmann[1], Ekaterina Demianchuk[1], Michelle Kaczmarski[1], Eric Sobierajski[1], Christian Riedel[1], Petra Wahle[1]

[1] Developmental Neurobiology, Faculty of Biology and Biotechnology, Ruhr University Bochum, Universitätsstraße 150, 44780 Bochum, Germany.

[§] equal contribution

*Corresponding author: Ina Köhler, Developmental Neurobiology, Faculty of Biology and Biotechnology, Ruhr University Bochum, Universitätsstraße 150, 44780 Bochum, Germany.
Email: ina.koehler@ruhr-universitaet.de

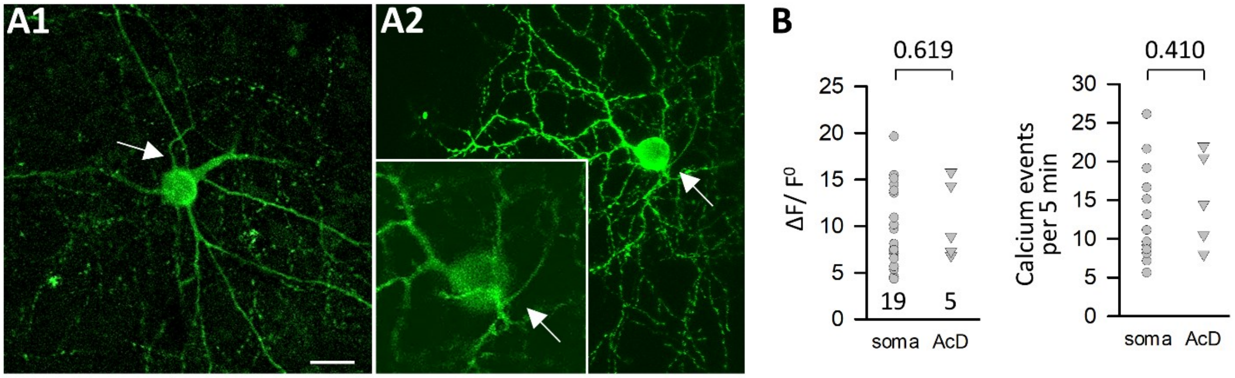

Supplementary Fig. 1 Activity of interneurons with somatic axons and axons originating from dendrites.

The cells recorded in Fig. 1E had been fixed and stained against the EGFP motif in GCaMP6f to classify the cell type and to separate cells by axonal origin from soma or an axon-carrying dendrite (AcD). A) representative cells with somatic axon (A1) and axon from dendrite (A2). In some cells, axons were already recognizable by GCaMP6f fluorescence at the peak of the calcium event. B) Calcium event frequency and amplitude were similar. Scale bar: 20 µm, arrows indicate axons.

Supplementary Table 1 Morphometry of pyramidal cells.

Table reports the mean ± S.E.M. of apical dendritic length and segments, and basal dendritic length and segments (the average per neuron) of the reconstructed pyramidal cells of L2/3 and L5/6 at DIV 10 and DIV 20. ANOVA on Ranks (AoR) versus control and Mann-Whitney Rank Sum test; p-values are given, significant differences in bold.

	**Pyramidal cells in L2/3**		**Pyramidal cells in layers L5/6**	
Age, Condition<br>(Number of batches)	ADL (n)<br>Segments	BDL<br>Segments	ADL (n)<br>Segments	BDL<br>Segments
**DIV 10**<br>Control (6)	1539 ± 74 (58)<br>31.7 ± 1.5	391 ± 17<br>8.8 ± 0.4	1328 ± 71 (34)<br>26.9 ± 1.8	355 ± 28<br>7.8 ± 0.5
CIQ	1513 ± 85 (27)<br>30.7 ± 1.8	354 ± 26<br>8.7 ± 0.6	1230 ± 97 (25)<br>25.0 ± 2.2	360 ± 33<br>8.1 ± 0.6
DQP	1549 ± 75 (47)<br>30.7 ± 1.5	361 ± 21<br>8.7 ± 0.5	1491 ± 64 (48)<br>31.8 ± 1.7	336 ± 23<br>8.3 ± 0.5
Anova on Ranks vs.<br>control	*0.911*<br>*1.000*	*0.565*<br>*0.979*	*0.045*<br>***0.019***	*0.875*<br>*0.865*
Mann-Whitney test<br>Control vs. CIQ			0.276<br>0.419	
Mann-Whitney test<br>Control vs. DQP			0.103<br>**0.036**	
**DIV 20**<br>Control (4)	1835 ± 104<br>(40)<br>29.8 ± 1.7	384 ± 26<br>7.6 ± 0.5	1758 ± 158<br>(28)<br>33.1 ± 2.7	351 ± 34<br>7.0 ± 0.6
CIQ	1694 ± 76 (36)<br>28.8 ± 1.6	402 ± 26<br>7.3 ± 0.5	1824 ± 93 (39)<br>33.2 ± 2.0	400 ± 23<br>7.8 ± 0.5
DQP	2032 ± 116<br>(36)<br>37.7 ± 2.1	362 ± 24<br>8.0 ± 0.6	1721 ± 154<br>(27)<br>32.8 ± 3.3	389 ± 33<br>8.2 ± 0.6
Anova on Ranks vs.<br>control	*0.119*<br>***0.001***	*0.520*<br>*0.695*	*0.420*<br>*0.825*	*0.222*<br>*0.151*
Mann-Whitney test<br>Control vs. CIQ	0.549			
Mann-Whitney test<br>Control vs. DQP	**0.003**			

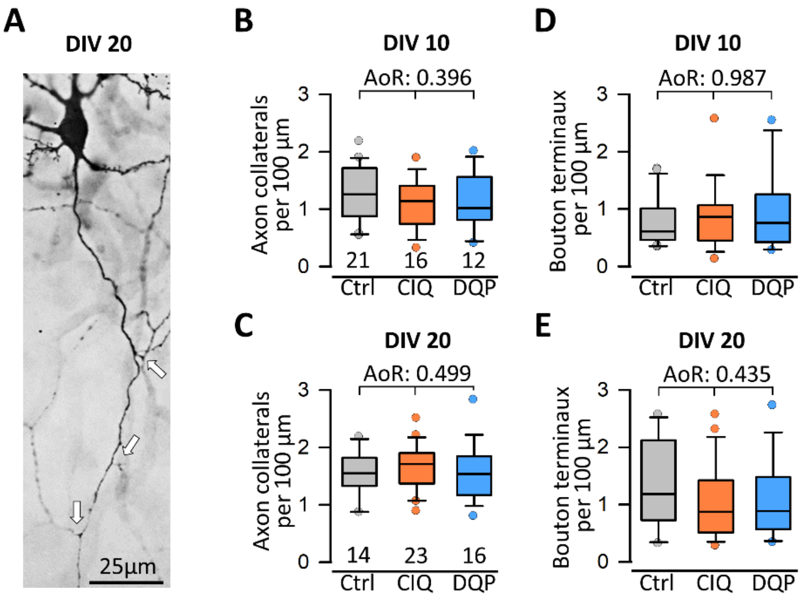

Supplementary Fig. 2 GluN2D signaling does not influence pyramidal cell axon collateralization.

A) Representative pyramidal cell with descending axon giving rise to oblique ascending thin collaterals (arrows; montage composed in Photoshop). B, C) Number of axon collaterals determined by plotting 500 µm of the primary descending main axon of L2/3 pyramidal cells, counting the branch points at DIV 10 and 20. D, E) Number of bouton terminaux along the primary descending main axon of L2/3 pyramidal cells at DIV 10 and 20 determined for the same cells plotted in (H, I). Note some more collaterals and more bouton terminaux at DIV 20, indicating the expected developmental increase albeit not influenced by GluN2D signaling. ANOVA on Ranks (AoR) versus control, p-value given above the boxplots. The n is given in the graphs.

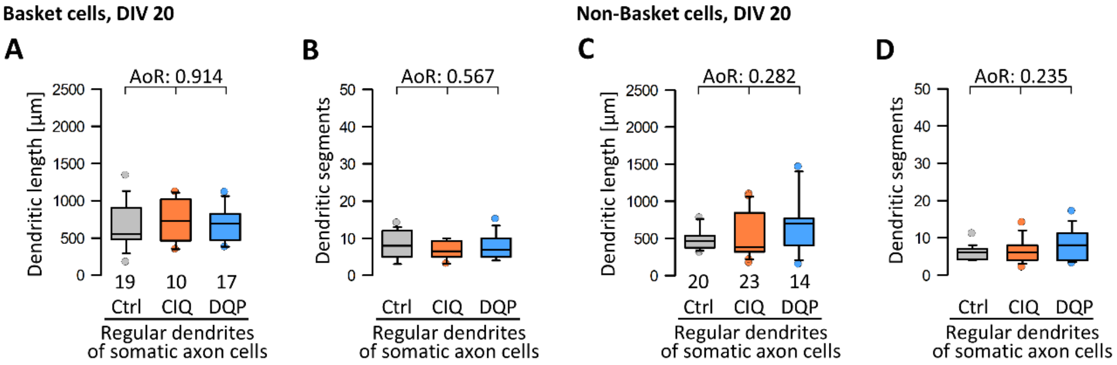

Supplementary Fig. 3 Dendritic parameters of interneurons at DIV 20.

A, B) Dendritic length and segments of basket cells. C, D) Dendritic length and segments of non-basket cells. Only cells with somatic axons have been considered to check if the treatment could alter dendrites at the later time window. The number of cells is between 10-23 (given in the graphs), and not even statistical trends were emerging. ANOVA on Ranks (AoR) versus control, p-value given above the box plots.

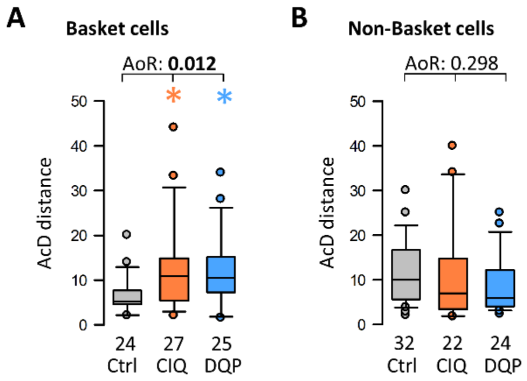

Supplementary Fig. 4 Basket cell AcD length is associated with longer distances between soma and axon origin.

A) Distance between soma and the point of origin of the axon from the AcD of basket cells. B) Distance between soma and the point of origin of the axon from the AcD of non-basket cells. ANOVA on Ranks (AoR) versus control; p-value given above the box plots. Significant p-values in bold, colored asterisks indicate the significant differences in the box plots.

Supplementary Table 2 Summary of protein blot analysis.

Cultures were treated DIV 5-10 and DIV 15-20, harvested 3 h (DIV 10) or 6 h (DIV 10, DIV 20) after the last pulse of CIQ or DQP. Mann-Whitney rank sum test versus control, significant p-values in bold. The number of lysates is reported; cultures were from >10 independent preparations.

Supplementary Table 2. Quantitative results of Western blot analysis of DQP or CIQ stimulated cultures at DIV10 and DIV20.

Age, Condition	GluN2B	GluN2A	GluN2D	Synapsin-1	p38	PSD-95	Syt-2	GAD-65	GAD-67	VGAT	$GABA_AR$ α1	Kv3.1b	GFAP
**(A) DIV 10, 3h**													
Control	1.000 ± 0.019 (10)	1.000 ± 0.022 (11)	n d.	1.000 ± 0.020 (7)	1.000 ± 0.029 (12)	1.000 ± 0.017 (14)	1.000 ± 0.016 (19)	1.000 ± 0.021 (13)	1.000 ± 0.030 (13)	1.000 ± 0.028 (7)	1.000 ± 0.016 (26)	1.000 ± 0.028 (9)	n d.
DQP	1.024 ± 0.028 (11)	1.030 ± 0.041 (9)	n d.	1.071 ± 0.024 (8)	0.993 ± 0.040 (12)	1.010 ± 0.016 (15)	0.973 ± 0.016 (20)	1.032 ± 0.032 (15)	1.063 ± 0.038 (15)	1.054 ± 0.029 (7)	1.012 ± 0.017 (27)	0.994 ± 0.019 (8)	n d.
Mann-Whitney test	0.647	0.543		0.082	0.977	0.844	0.156	0.549	0.549	0.208	0.950	0.885	
Control	1.000 ± 0.019 (10)	1.000 ± 0.022 (11)	n d.	1.000 ± 0.020 (7)	1.000 ± 0.029 (12)	1.000 ± 0.017 (14)	1.000 ± 0.016 (19)	1.000 ± 0.021 (13)	1.000 ± 0.030 (13)	1.000 ± 0.028 (7)	1.000 ± 0.016 (26)	1.000 ± 0.029 (12)	n d.
CIQ	1.036 ± 0.038 (11)	1.070 ± 0.057 (11)	n d.	1.052 ± 0.030 (8)	1.002 ± 0.032 (13)	1.011 ± 0.021 (15)	1.004 ± 0.027 (20)	1.022 ± 0.029 (15)	1.041 ± 0.041 (15)	1.059 ± 0.033 (8)	0.987 ± 0.017 (27)	**1.109 ± 0.039 (13)**	n d.
Mann-Whitney test	0.805	0.293		0.232	0.807	0.983	0.725	0.818	1.000	0.281	0.637	**0.024**	
**(B) DIV 10, 6h**													
Control	1.000 ± 0.019 (26)	1.000 ± 0.021 (23)	1.000 ± 0.015 (9)	1.000 ± 0.014 (21)	1.000 ± 0.026 (46)	1.000 ± 0.033 (15)	1.000 ± 0.020 (24)	1.000 ± 0.025 (35)	1.000 ± 0.026 (35)	1.000 ± 0.013 (51)	1.000 ± 0.022 (25)	1.000 ± 0.012 (33)	1.000 ± 0.022 (7)
DQP	**0.925 ± 0.020 (25)**	0.970 ± 0.028 (24)	1.041 ± 0.069 (9)	1.005 ± 0.013 (21)	0.958 ± 0.023 (46)	0.963± 0.020 (15)	**0.908 ± 0.022 (24)**	**0.894 ± 0.020 (34)**	0.967 ± 0.017 (34)	**0.940 ± 0.014 (50)**	0.975 ± 0.020 (26)	0.990 ± 0.016 (33)	0.981 ± 0.044 (6)
Mann-Whitney test	**0.006**	0.292	0.930	0.940	0.137	0.340	**0.014**	**< 0.001**	0.288	**0.003**	0.332	0.349	0.836
Control	1.000 ± 0.014 (39)	1.000 ± 0.016 (36)	1.000 ± 0.013 (10)	1.000 ± 0.012 (16)	1.000 ± 0.024 (59)	1.000 ± 0.015 (16)	1.000 ± 0.018 (50)	1.000 ± 0.019 (51)	1.000 ± 0.020 (51)	1.000 ± 0.012 (57)	1.000 ± 0.015 (39)	1.000 ± 0.012 (33)	1.000 ± 0.022 (7)
CIQ	1.010 ± 0.016 (40)	1.009 ± 0.024 (35)	1.038 ± 0.036 (10)	1.010 ± 0.019 (16)	0.961 ± 0.021 (60)	1.014 ± 0.019 (17)	0.977 ± 0.017 (50)	**0.891 ± 0.016 (51)**	0.960 ± 0.016 (51)	**0.928 ± 0.013 (55)**	0.976 ± 0.016 (41)	0.980 ± 0.013 (33)	1.016 ± 0.048 (6)
Mann-Whitney test	0.663	0.523	0.850	0.720	0.365	0.825	0.365	**< 0.001**	0.060	**< 0.001**	0.312	0.147	0.836
**(C) DIV 20, 6h**													
Control	1.000 ± 0.045 (11)	1.000 ± 0.024 (6)	n d.	n d.	1.000 ± 0.021 (19)	1.000 ± 0.036 (6)	1.000 ± 0.024 (10)	1.000 ± 0.016 (19)	1.000 ± 0.021 (19)	1.000 ± 0.020 (15)	1.000 ± 0.021 (15)	1.000 ± 0.023 (9)	n d.
DQP	0.980 ± 0.044 (10)	0.946 ± 0.037 (7)	n d.	n d.	1.014 ± 0.031 (20)	1.057 ± 0.028 (6)	**0.913 ± 0.024 (9)**	**0.932 ± 0.014 (20)**	0.979 ± 0.018 (20)	1.008 ± 0.013 (16)	0.957 ± 0.024 (16)	0.986 ± 0.019 (10)	n d.
Mann-Whitney test	0.699	0.653			0.643	0.394	**0.025**	**0.002**	0.423	0.707	0.244	0.653	
Control	1.000 ± 0.045 (11)	1.000 ± 0.024 (6)	n d.	n d.	1.000 ± 0.021 (19)	1.000 ± 0.036 (6)	1.000 ± 0.024 (10)	1.000 ± 0.016 (19)	1.000 ± 0.021 (19)	1.000 ± 0.020 (15)	1.000 ± 0.021 (15)	1.000 ± 0.023 (9)	n d.
CIQ	0.965 ± 0.022 (11)	1.051 ± 0.052 (7)	n d.	n d.	1.045 ± 0.031 (20)	1.000 ± 0.027 (6)	0.980 ± 0.042 (9)	**0.918 ± 0.016 (20)**	0.992 ± 0.016 (20)	0.980 ± 0.019 (16)	0.975 ± 0.022 (16)	1.051 ± 0.026 (10)	n d.
Mann-Whitney test	0.237	0.365			0.267	0.818	0.653	**0.002**	0.684	0.514	0.441	0.178	
